# Does youth participation in the farming program impact farm productivity and household welfare? Evidence from Nigeria

**DOI:** 10.1016/j.heliyon.2023.e15313

**Published:** 2023-04-17

**Authors:** Abdulrazaq K. Daudu, Tahirou Abdoulaye, Zoumana Bamba, Suleiman B. Shuaib, Bola A. Awotide

**Affiliations:** a Department of Agricultural Extension and Rural Development, University of Ilorin, Ilorin, Nigeria; b Social Science and Agribusiness, International Institute of Tropical Agriculture (IITA), Bamako, 91094, Mali; c Department of Science Education, Agriculture Unit, University of Ilorin, Ilorin, Nigeria; d Country Representative, International Institute of Tropical Agriculture (IITA), Kinshasa 4163, Congo

**Keywords:** Agriculture, Youth employment, Household welfare, Propensity score matching, Endogenous regression, Northern Nigeria, Participation

## Abstract

One significant issue in the Global South, particularly in sub-Saharan Africa, is youth unemployment. This is a result of the aging and shrinking agriculture industry, increased unemployment rates mixed with a sizable unskilled workforce, and rapid population expansion. Studies have shown that farming programs, when supported by increased investment and regulatory frameworks, provide opportunities for meaningful employment for many young people. This study attempts to estimate the impact of youth participation in farming programs on farm productivity and welfare using rice-growing household data from northern Nigeria. We used propensity score matching (PSM) and endogenous switching regression (ESR) to address biases that may arise from both observed and unobserved factors. Our results show that age, education, household size, farm size, extension, access to credit, and membership of a social group are positive and significantly associated with youth participation in farming programs. The outcome demonstrates that participants fare better than non-participants in terms of farm productivity and welfare status. Furthermore, youth participation in farming programs has implications for heterogeneity within the participant group, which depends on socio-economic characteristics such as access to finance, association membership, and education, emphasizing the need for specific interventions and focusing on particular youth groups. Therefore, access to credit through relevant agencies with low interest rates and flexible payment options, strengthening youth organization could encourage participation in farming programs and job opportunities for the prosperity of the rural economy.

## 1 Introduction

The youth surplus is particularly visible in sub-Saharan Africa, home to ten of the world's youngest countries, and where this trend is likely to accelerate in the coming decades [1]. Young populations, especially in emerging economies, can be cheap. Between 2011 and 2030, the World Bank expects Africa's population change to deliver 11–15% GDP growth [1]. However, this expansion is dependent on countries with young populations that offer their residents adequate education, training and job opportunities, and failure to do so could be dangerous, as “a lack of meaningful job among young people has in certain instances led to social upheaval, insecurity, or unmanaged migration” [2]. When it comes to earning a living, youth in most developing nations, including Nigeria, confront numerous obstacles. The National Policy on Youth Development in Nigeria defines youth between the ages of 18 and 35 [3], with the majority of these youth (both male and female) living in rural areas with limited opportunities for productive employment [4]. But because of their youth, resourcefulness and entrepreneurial spirit, they have an undeniable and untapped potential to transform the agricultural sector [5].

Nigeria also has one of the largest youth populations in the world, with over 13.9 million unemployed Nigerian youth as of the second quarter of 2020. Unfortunately, the unemployment rate is rising in parallel with the growth in the youth population. According to NBS, Nigeria's youth population is estimated at 40 million, of which only 14.7 million are in full-time employment and another 11.2 million are unemployed [6]. Agriculture can provide productive employment opportunities for large numbers of young people, provided it is supported by increasing investment and a favorable legal and policy environment [[7], [8], [9]]. However, the youth are rapidly turning away from farming in search of lucrative and business-oriented occupations. While Nigeria is grappling with the problem of unemployment among the country's teeming youth [10], the country's successful government has designed and implemented agricultural development programs to enhance the economic potential of youth over the years [11]. These programs include the National Directorate of Employment (NDE), which was established in 1986 to provide micro credit to youth who wish to start their own businesses and eventually become self-employed [12]. In addition, the New Nigeria Agricultural Policy (NNAP) was developed in 2001 with the aim of ensuring food security through the introduction of better seeds and recognizing the potential of young and small farmers as main food suppliers [13], the Nigerian Incentive-Based Risk Sharing System for Agricultural Lending (NIRSAL), launched in 2010, which encourages farmers to insure their farms against natural disasters and take out loans from commercial banks that guarantee their loans. However, the program has been hampered by political inconsistencies and a lack of participation from farmers and young adults [14]. More recently, in 2015, the Nigerian federal government, through the Central Bank of Nigeria (CBN), established the Anchor Borrowers' Program (ABP) in line with its development objective to provide a long-term solution to some of the shortcomings of previous programs and to Lowering the country's unemployment rate. This ABP was instituted to reduce the government's excessive costs associated with importing staple foods, particularly rice, that can be locally produced and to create employment opportunities for Nigerian youth.

The impact of youth participation in farming programs on farm productivity and household welfare has not been examined in the growing empirical literature on youth participation in farming, nor in numerous impact assessments. For example, most farming programs in Nigeria are structured and designed to provide employment for young people; however, there is little research on the impact of youth participation in farming programs [15,16]. This points to a significant gap in the literature that our study aims to fill. Therefore, this study fills this vacuum by presenting empirical data on the effects of youth participation in farming on outcome variables and using quantitative evidence from a cross-sectional dataset. Such empirical evidence could inform policies in sub-Saharan Africa aimed at rural youth development and unemployment. Numerous research, like those by Kassie et al. [17] and Ahmed et al. [18], have employed an economic model like Propensity Score Matching (PSM) to evaluate program impact. PSM is the best option when differences between participants and non-participants can only be explained by observable features. However, PSM results are subject to bias, particularly when factors such as motivation, farming skills, social networks, and informal connections are present. We use the Endogenous Switching Regression (ESR) model [[18], [19], [20], [21], [22]] to address these issues and improve the majority of previous studies. This model accounts for both observable and unobservable confounders that influence rural youth's decision to participate in agriculture (rice cultivation). The following are the primary policy issues addressed in this study: (1) What attributes influence rural youth's decision to engage in agriculture? (2) does participation of rural youth in farming (rice cultivation) increase farm productivity and household welfare? Answering these questions is central to advancing action to tackle youth unemployment, improve farm yields and their welfare in Nigeria and sub-Saharan Africa. It is equally important to remember that farming is essential to the most emerging nations' economies and has been associated in SSA, with improving wealth [23].

This study will no doubt contribute to the growing body of organized knowledge about youth participation in farming and, in terms of policy, help formulate economic policies to make farming business more attractive to the country's teeming youth, the rural-urban-limit migration and maintain food security. Our study attempted to recommend the characteristics or factors that should be incorporated into agricultural policies to improve farm productivity and household well-being through rural youth participation in farm operations.

The remainder of the work is structured as follows: the second section dealt with relevant literature on youth participation in farming, the third section covers the study area, the survey design, the methods and the empirical model, the fourth section reports the results of the main findings and discussions and finally the fifth section draws conclusions from the results.

## 2 An overview of the anchor borrowers programme (ABP)

In an effort towards improving agricultural development and attaining self-sufficiency in food production in the country, the Central Bank of Nigeria (CBN) launched Anchor Borrowers' Programme (ABP) with its pilot phase in Kebbi State in (2015). The ABP aims at creating economic linkages between over 600,000 smallholder farmers (out-growers) and reputable large-scale processors (off-takers) with a view to increasing agricultural output and significantly improving capacity utilization of integrated mills. According to the CBN (2016), the fall in oil prices has given Nigeria a timely reminder that the country has no choice but to diversify its economy away from oil, and into agriculture, manufacturing, services, and other non-oil sectors. From the CBN guide on the ABP, Nigeria's agricultural commodities and food import bill has averaged over N1trillion in the past two years. Food products like milk, sugar, rice, wheat and fish accounted for N901billion or 93.5% and N788billion or 88.71% of this total in 2013 and 2014, respectively. These figures are exclusive of the activities of smugglers. The import bill of rice and wheat was estimated at N428billion in 2013 and N307billion in 2014. These huge amounts were expended on items that the country has the potential to produce locally with the attendant loss of employment generation and wealth creation opportunities.

Furthermore, the allocation of foreign exchange to the importation of these items has continually depleted our foreign reserve, which has been on a steady decline in recent times. The current effort of the CBN to stimulate local production of the commodities is largely due to the adverse effect of their importation to the nation's foreign reserves. Under the intervention, the CBN has set aside the sum of N20billion from the N220billion Micro, Small and Medium Enterprises Development Fund (MSMEDF) for farmers at a single-digit interest rate of 9%. The programme seeks to pursue objectives such as, creation of jobs, reduction in food imports and diversification of the economy. Also, the CBN guaranteed half the value of any loan defaults. The anchors have access to some grants and wavers. Anchors sign agreements with smallholder farmers in which they supply input in exchange for guaranteed sales of a proportion of the crop (usually 80%) at a pre-agreed price, with the cost of inputs deducted from these sales. Farmers are expected to organize themselves into co-operatives and to engage in cross-guarantees. About 30 large enterprises have expressed their interest in the ABP. The government facilitates technical services, certification and minimization of the risk of contracts failing to be honoured. The out-grower scheme also includes plans to facilitate land title registration in a second phase. Over the years, the non-availability of off-takers or favorable market for farmers' products, which often results in low price offered for the products, tends to discourage farmers from expanding production. There has also been a failure in past credit schemes to link farmers and potential buyers. As a result, there is the need therefore for research to investigate the impact of any further lending scheme established by Nigeria government, with a view to deriving policy for better performance.

## 3 Review of relevant literature

A growing body of literature is emphasizing the value of youth participation in farming business in emerging economies like Nigeria. An example is a study by Ref. [16] on the technical efficiency of youth participation in the farming program in Ondo State, Nigeria, in which they found different levels of technical efficiency among youth. The primary factors influencing youth production performance in the program, according to the authors, are land, labor, agrochemicals, and planting material. Youth participation, according to Checkoway [24], is the active influence and involvement of young people. This depends not only on their symbolic roles or inactive engagement in adult organizations, but also on how well-qualified they are, e.g., if they actually influence the process, shape a certain decision or lead to a positive consequence [25]. While youth participation is essential for a country's economy to thrive, there are additional socio-economic barriers that prevent youth from engaging in farming business [25]. Parents can discourage their children from pursuing careers in farming [26] or encourage them to choose salaried employees [27], which purportedly offer greater financial rewards and fewer elements of risk [28]. Furthermore, the need to meet basic needs immediately, lack of economic choice or certainty of inheriting the land influence the decision to participate in farming for the majority of young people in rural areas [29]. Tiaraiyerari and Krauss [30] assessed young people's perceptions of Malaysia's urban farming initiative. The authors found that optimism about farming, career motives, support from family and friends, and enthusiasm for farming had a significant and positive impact on young people's participation in the program. On the other hand [31], found that while young people in Ghana found agriculture profitable, many left the industry because they lacked access to vital agricultural resources such as land and finance. Etim and Udo [32] examined the willingness of young people in Nigeria to engage in farming. The results showed that age, young people's farming experience, household income, and membership of a social group were all significant positive influences on young people's willingness to engage in farming. Similarly [33], examined the willingness of unemployed Nigerian graduates to engage in agriculture to address the problem of youth unemployment. The authors observed that factors such as gender, education, participation in farm training programs, and marital status had a significant impact on young people's propensity to participate in farming business. Certain research papers [15,34,35] have investigated the variables influencing youth participation in farming in Nigeria.

According to the results of these studies, the factors that promote the participation of young people in farming activities include age, frequency of consultation visits, affiliation with a social organization, household size and farm size. According to a study by Ref. [36], insufficient incentives, lack of farming education, limited agricultural knowledge, limited access to finance and poor agricultural prospects are some of the barriers that prevent young people from working in agriculture. Compared to other population groups [37], found that fewer young people are engaged in farming. According to the study by Ref. [38], optimism has increased that farming will offer young people opportunities for sustainable living. Similar to Maritim et al. [39], youth participation in farm operations in Kericho County, Kenya was significantly and favourably influenced by access to credit, land, and perceived rewards. Furthermore [40], found that exposure to farming skills at various educational levels had an impact on young people's intentions to engage in agripreneurship. This emphasizes the significance of encouraging agricultural education at various educational levels, boosting awareness of farming as a career alternative, and ultimately increasing youth involvement in farming. Young people's involvement in farming activities becomes more likely when youth capacity is increased through education and other programs and this capacity is then used to improve their socio-economic access to industrial actors in the agricultural sector [41,42]. This would empower young people and equip them with the skills they need to participate in the dynamic global agricultural value chains [43]. Although difficulties caused by the lack of mainstream services in rural areas can discourage young people from engaging in farming and thriving [43]. They may be responsible for their limited participation and unfavorable views on agriculture. Undoubtedly, young people's difficulties in accessing useful resources and services in rural areas also contribute to low participation [25].

## 4 Methodology

### 4.1 Description of study area

**Fig. 1:**
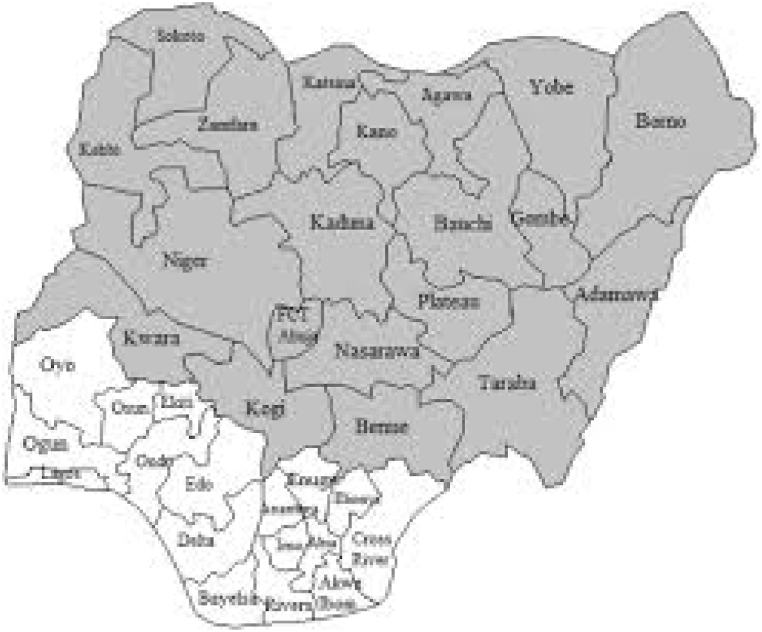
Maps of Nigeria showing the study areas (Northern region).

Source: Author (2021)

The study was conducted in the northern region of Nigeria. It comprised the state of Bauchi in the northeast, the state of Benue in the northcentral and the state of Kebbi in the northwest. These areas were selected primarily for their active participation in the Anchor Borrower Scheme (a program that encourage youth participation in farming business). Until 1976, Bauchi State was a province in what was then the north eastern state of Nigeria. According to the 2006 census, the state had a population of 4,653,066. The capital is the city of Bauchi. It occupies a total area of 49,119 km^2^, which is about 5.3% of the total land mass of Nigeria. Bauchi state is one of the states in northern Nigeria that includes two distinct vegetation zones namely the Sudan savanna and the Sahel savanna. Benue State, located in the north-central region of Nigeria, has a total population of 4,253,641 as of the 2006 census with an average population density of 99 persons per km^2^. Benue State is located in the lower Benue River Valley in the mid-belt region of Nigeria. Its geographic coordinates are longitude 7° 47′ and 10° 0′ east and latitude 6° 25′ and 8° 8′ north. Benue occupies a land mass of 34,059 square kilometers. It is the nation's acclaimed food basket as farming is the mainstay of the economy, employing over 75% of the state's farming population. Its rich agricultural products such as yam, rice, beans, cassava, sweet potato, corn, soybean, sorghum, millet, sesame, cocoyam, etc. The state of Kebbi is located at the coordinates 11°30′N 4°00′E and has a total population of 3,137,989 as per the 2006 census, in 21 local administrative areas. Agriculture is the main occupation of the people, especially in rural areas. The crops grown are mainly cereals; Animal husbandry and fishing are also common. The state of Kebbi has become one of the largest rice producers in Nigeria. The current involvement of more than 70,000 farmers in rice production and making Kebbi a new center for agro-based commodities in the country. The map of Nigeria showing the study locations is presented in Fig. 1.

### 4.2 Data and sampling methods

We used data from the household rice cultivation survey conducted between November 2020 and January 2021 as part of the Enhancing Capacity to Apply Research Evidence (CARE) policy on youth engagement in agribusiness and rural economic activities in Africa was funded by the International Fund for Agricultural Development (IFAD) and the International Institute of Tropical Agriculture (IITA) in Nigeria. The data collection process in the survey involved a multi-level stratified random sampling technique from a cross-section of rural youth who grow rice either as a primary or secondary occupation. The first stage involved a targeted selection of one state from each of the three regions in northern Nigeria based on their active participation in rice cultivation under the Anchor Borrowers Program (ABP). These states include: Bauchi (Northeast), Benue (Northcentral), and Kebbi (Northwest). In the second stage, with all local governments participating in the 3 selected states, 25% of the local governments (LGAs) with the highest youth participation in rice cultivation were targeted from 20 LGAs in Bauchi State, 23 LGAs in Benue State, and 21 LGAS from the State of Kebbi, making a total of approximately 16 LGAs (i.e., 5 from Bauchi, 6 from Benue and 5 from Kebbi States). The list of rice-growing communities was obtained from the Department of Agriculture of each selected LGA. In stage three; 2 rice farming communities from each of the sampled LGAs were randomly selected, a total of 32 rural farming communities for the study. Finally, the fourth stage involved a random selection of 15 young rice farmers participating in ABP and 15 non-participating young rice farmers in ABP from each of the selected farming communities, resulting in a total of 960 respondents for the study. Although only information from 932 of the total sampled respondents was eventually deemed worthy and used for the study, resulting in a 97.1% success rate.

We collected data using a structured questionnaire delivered on an Android electronic tablet software (Surveybe). The survey questionnaire was segmented according to the objectives of the study. Information was collected from respondents on the socio-economic characteristics of farm households, rice production, expenditure on food and other items, income from rice cultivation, and the decision to engage in farming business (rice cultivation). Consent from respondents was obtained prior to answering the enumerators' questions. All respondents were asked not to participate in the study if they felt uncomfortable and to withdraw at any time during the survey. Each respondent would be informed in an appropriate manner about the aim of the study and the importance of participating in the study. Respondents were required to sign a consent form before answering the narrators' questions, and if at any point during the survey they felt uncomfortable, all participants were encouraged to stop the survey. We adopted three main variables for welfare in this study and these include the size of rice farm yield in hectares, food expenditure and total household expenditure (non-food). The size of the rice crop harvested in the last year of the farming season, measured in tons per hectare of cultivated area. Food expenditure was measured by asking sample households about food spending for the previous survey year, which covered 12 months in accordance with the World Bank's LSMS-ISA standard module. Following similar impact studies [44,45], we measured total household expenditure per capita by dividing total household expenditure by the number of household members. Our treatment variable is rural youth's decision to participate in farming (rice growing) under the ABP program, measured by a dummy that takes a value of 1 when a rural youth chooses to participate in farming (rice growing) decided, and 0 otherwise. For this study, we used the propensity score matching method (PSM) and the endogenous switching regression (ESR) model to compare the relationship between the outcome variables (farm productivity and welfare) and the exogenous evaluate variables. The endogenous switching regression (ESR) model was used to account for both observable and unobservable confounders affecting youth participation decision making in farming business [44,[46], [47], [48]].

### 4.3 Empirical estimation techniques

The empirical evidence from recent studies on rural youth's choices to engage in farming business provides guidance for the choice of variables to include in our model. Rural youth's decision to engage in farming could be viewed in the context of general utility maximization. In this case, rational rural youth will only continue to participate in farming (rice cultivation) if their participation increases their farm yield and income, and invariably translates into improved household welfare. Assume the treatment indicator ${Ρ}_{i}$ represents the decision to engage in farming or not by rural youth, which is thought to depend on farm productivity and perceived household utility. However, given that participation in farm business (rice cultivation) is likely to have an impact on farm productivity and household welfare outcomes, these outcomes form a linear function of the vector of explanatory variables observed by rural youths, including the dummy variables explaining their choice of participating in farming (rice cultivation), which could be expressed in Equation (1) as:

(1) $${Α}_{i}=\varphi {Β}_{i}+\varphi {Ρ}_{i}+\eta_{i}$$

where ${Α}_{i}$ presents farm productivity and welfare outcomes; ${Β}_{i}$ represents the explanatory variable that includes socio-economic characteristics, production and institutional variables of rural youth; ${Ρ}_{i}$ is the treatment indicator for decisions to involve young people from rural areas; φ is the parameter vector of the explanatory variable to be estimated; and φ the associated parameter measures the impact of rural youth participation in farming (rice cultivation) on farm productivity and household welfare variables; and $\eta_{i}$ is the error term. In the absence of a non-random allocation of the rural youth to the treatment (participant) and control group (non-participant), the participation of the youth in farm business (rice cultivation) is denoted by ${Ρ}_{i}$ is potentially endogenous, making it difficult to identify its impact on outcome variables. The identification challenge arises from the fact that rural youth's decision to engage in farming (rice cultivation) might be based on unobservable quantities that correlate with both outcomes and observable predictors. For example, the agronomic farming skills of young rural farmers cannot be observed but can affect both farm yields and their household welfare. Therefore, ignoring the potential endogenous selection bias in the outcome equations can lead to biased and inconsistent estimators.

According to recent studies by Refs. [44,45,47,[49], [50], [51], [52], [53], [54]], we apply semi-parametric (propensity score matching, PSM) and parametric (endogenous switching regression model, ESR) approaches that take into account the potential self-selection problem. The latter is used as a robustness check for the results of the former. According to its observable characteristics, the PSM technique estimates propensity values, which are estimates of rural youth's propensity to engage in farming (rice cultivation). This strategy eliminates selective bias by mimicking the randomized control strategy. In addition, it is possible by matching methods to determine the parameters of the average treatment effect (ATE) and the average treatment on treated (ATT). The ATE and ATT differ in that the former estimates the mean improvement across all study participants, while the latter estimates the impact of differences in the subpopulation of rural youth engaged in farm business (rice cultivation); In other words, the ATT estimates how farm yield and the welfare of rural youth would have changed if participants had decided not to grow rice.

In our investigation, we use the propensity score matching (PSM) approach, and the key steps in using the PSM in the present study are (a) estimating propensity scores of youth participation in farming (rice cultivation) using of the logit model, (b) imposition of a common support region, (c) matching of slope values between the participating group and the non-participating group of rice cultivation in the common support region using different algorithms, such as nearest neighbor (NNM), and kernel-based matching (KBM) and (d) assessing the quality of matches and (e) estimating the impact. However, the NNM and KBM methods are the simplest and most widely used matching methods. In the NNM, the untreated individuals are selected as partners who closely match the propensity score of the treated individuals. Kernel matching takes information from all non-participants and constructs the counterfactual results using a weighting function and reduces the variance [55]. The parameters of interest in this study are the ATT (see Equation (2)), which allows us to assess farm productivity and welfare of rural youth participants engaged in farming (rice cultivation) if they had chosen not to be in the treatment group. According to Ref. [56] the ATT is expressed in Equation (2) as follows:

(2) ATT=E[Y(1)−Y(0)|T=1]………………….

where *Y* (1) and *Y* (0) are outcome indicators for the value of farm yield per hectare and welfare (measures as total household expenditure per capita and food expenditure per capita). *T* represents the treatment indicator, which is equal to 1 for participants. However, the non-participants status of participants cannot be observed E[Y(1)|T=0]; we can only observe E[Y(1)|T=1]. To observe the counterfactual status of the treated group, the PSM constructs a comparable counterfactual picture of rural youth for the treated group from the already matched observable characteristics of populations, based on the assumption that there is no systematic difference between their non observable features. Given the satisfaction of the conditional independence assumption, represented as *TY*1, *Y*0 ⊥*T*/*B*, meaning that their farm productivity and welfare outcomes are dependent on observable characteristics of rural youth independent of participation in farming (rice cultivation), and the overlap condition, which ensures that the treatment observation has adjacent comparison observations in the propensity score distribution and inferences about causality can only be made in areas of common support, then the ATT will be computed using Equation (3) given as:

(3) ATT=E[Y(1)|T=1,p(x)]−E[Y(0)|T=0,p(x)]

However, the PSM is limited only to evaluating observational data from rural youth in generating ATT estimates, which may be biased due to the assumption that there are no differences in the unobservable characteristics of rural youth. In reality, differences in farm productivity and welfare outcomes may depend on unobservable innate skills and motivations that might influence rural youth's decision to participate in farming business (rice cultivation). For a more robust approach and consistent estimation of the impact of youth participation in farming (rice cultivation) on the outcomes of interest (farm productivity and welfare), we considered using the linear endogenous switching regression model that accounts for both observed and unobserved sources bias [44,[46], [47], [48]]. The switching regression model is a variant of the classic Heckman selection model. ESR has two equations that are estimated simultaneously in STATA using the selection and result equations. The ESR approach addresses this endogeneity problem by simultaneously estimating the selection and outcome equations using Full Information Maximum Likelihood (FIML) [44,[46], [47], [48]]. Here we derived the selection equation by supposing that rural youth make participatory decisions about whether or not to participate in farming (rice cultivation) based on expected benefits (in terms of yield and welfare gains). Suppose a rational rural youth will choose to enter treatment if farm productivity and welfare gain are significantly reasonable, i.e., $\Upsilon_{i}^{*}=\mu_{1}-\mu_{0}$. Since $\Upsilon_{i}^{*}$ cannot be observed directly, it can be expressed in Equation (4) as a latent variable model as follows:

(4) $$\Upsilon_{i}^{*}=\alpha_{1}+\beta {Η}_{i}+\mu_{i}\;with\;T_{i}=\left\{ \begin{array}{c} 1\;if\;\Upsilon_{i}^{*}>0\\ 0\;if\;\Upsilon_{i}^{*}\leq 0 \end{array}\right.\ldots \ldots \ldots \ldots$$

whereby $\Upsilon_{i}^{*}$ is the latent variable indexing the likelihood of rural youth participating in farming (rice cultivation); ${Η}_{i}$ represents variables affecting expected benefits from participation in farming (rice cultivation); β is a vector of parameters to be estimated, and $\mu_{i}$ is a random error related to youth participation in farming (rice cultivation). The result function dependent on $\Upsilon_{i}^{*}$, can be specified as follows, where rural youth are faced with two regimes (1) participation and (2) non-participation, and then can be represented in Equations (5), (6), respectively as an ESR model in the following way.

(5) $$Regime\;1\;\left(participant\right):Y_{1i}=\beta_{1}S_{1i}+\varepsilon_{1i}\;if\;T_{i}=1\ldots \ldots \ldots \ldots \ldots \ldots$$

(6) $$Regime\;2\;\left(nonparticipant\right):Y_{2i}=\beta_{2}S_{2i}+\varepsilon_{2i}\;if\;T_{i}=0\ldots \ldots \ldots \ldots \ldots$$

where $Y_{1i}$ and $Y_{2i}$ represents the outcome indicator of treated vs. untreated rural youth; $S_{i}$ depicts a vector of exogenous variables affecting outcome variables; *β* is a vector of parameters to be estimated; $\varepsilon_{1i}$ and $\varepsilon_{2i}$ are the error terms linked to the outcome equations. According to Ref. [57], the error terms in the selection equation (4) and the outcome equations (5), (6) are assumed to have a triumvirate normal distribution with a mean of zero and a non-singular covariance matrix is given in Equation (7) as follows:

(7) $$Cov\;\left(\mu_{i},\varepsilon_{1i},\varepsilon_{2i}\right)=\left[\begin{array}{ccc} \sigma_{\mu }^{2} & \sigma_{{\mu \varepsilon }_{1}} & \sigma_{{\mu \varepsilon }_{2}}\\ \sigma_{{\mu \varepsilon }_{1}} & \sigma_{\varepsilon_{1}}^{2} & \sigma_{{\varepsilon_{1}\varepsilon }_{2}}\\ \sigma_{{\mu \varepsilon }_{2}} & \sigma_{{\varepsilon_{2}\varepsilon }_{1}} & \sigma_{\varepsilon_{2}}^{2} \end{array}\right]\ldots \ldots \ldots \ldots \ldots ..$$

where $\sigma_{\mu }^{2}$ = variance of the error term in the selection equation, which is assumed to be 1; $\sigma_{\varepsilon_{1}}^{2}$ and $\sigma_{\varepsilon_{2}}^{2}$ = variance of the error terms in the result equations. $\sigma_{{\mu \varepsilon }_{1}}$ and $\sigma_{{\mu \varepsilon }_{2}}$ = covariance of $\mu_{i},\varepsilon_{1i}\text{,}$ and $\varepsilon_{2i}$, which measure the direction and degree of non-random selection. The fact that the outcome variables $Y_{1i}$ and $Y_{2i}$ cannot be determined simultaneously [57], therefore no covariances between the error terms in the result equations $\sigma_{{\varepsilon_{1}\varepsilon }_{2}}$ and $\sigma_{{\varepsilon_{2}\varepsilon }_{1}}$ could be defined. Hence, Equations (8), (9) represent the expected values of the error terms $\varepsilon_{1i}$ and $\varepsilon_{2i}$ due to the respondent's participation criterion is non-zero because of the possible correlation between the error term in the participation equation and the error terms in the outcome equations.

(8) $$E\left(\varepsilon_{i1}|Y_{i}=1\right)=\sigma_{{\mu \varepsilon }_{1}}\left\{\frac{\theta \left(\hat{Y}\right)}{\varphi \left(\hat{Y}\right)}\right\}\ldots \ldots \ldots \ldots$$

(9) $$E\left(\varepsilon_{i2}|Y_{i}=0\right)=-\sigma_{{\mu \varepsilon }_{2}}\left\{\frac{\theta \left(\hat{Y}\right)}{1-\varphi \left(\hat{Y}\right)}\right\}\ldots \ldots \ldots \ldots$$

where θ(.) is the standard normal probability density function, φ(.) is the standard normal cumulative function; $-\frac{\theta \left(\hat{Y}\right)}{\varphi \left(\hat{Y}\right)}$ and $\frac{\theta \left(\hat{Y}\right)}{1-\varphi \left(\hat{Y}\right)}$ are the endogenous choice terms or inverse Mill's ratio weighted at $\hat{Y}={Η}_{i}\beta$ in the participation equation where $\hat{Y}$ is the predicted probability of rural youth involvement in farming (rice cultivation), $Y_{i}$. Because the ESR model treats the problem of selection bias as a missing variable problem, the inverse Mill's ratio terms from the probit model are included in the linear outcome Equations (10), (11) to correct for potential selection bias, which can be given as:

(10) $$Y_{1i}=\beta_{1}S_{1i}+\sigma_{{\mu \varepsilon }_{1}}\left\{\frac{\theta \left(\hat{Y}\right)}{\varphi \left(\hat{Y}\right)}\right\}+\in_{1i},if\;T_{i}=1\ldots \ldots \ldots \ldots \ldots \ldots$$

(11) $$Y_{2i}=\beta S_{2i}-\sigma_{{\mu \varepsilon }_{2}}\left\{\frac{\theta \left(\hat{Y}\right)}{1-\varphi \left(\hat{Y}\right)}\right\}+\in_{2i},if\;T_{i}=0\ldots \ldots \ldots \ldots \ldots \ldots$$

In addition to the above equations, which can be estimated in a two-stage procedure, the simultaneous estimation of the participation of rural young people in farming (rice cultivation) and outcome equations using the full information maximum likelihood (FIML) are therefore based on [48] could be considered an efficient approach. Therefore, FIML can be implemented in Stata using the movestay command. A statistically significant estimate of $\sigma_{{\mu \varepsilon }_{1}}$ and $\sigma_{{\mu \varepsilon }_{2}}$ indicates endogenous conversion. However, the model discussed is used to estimate the average treatment effect on the treated and the untreated, i.e., the ATT and ATU. The equations are given as follows:

For participants with participation:

(12) $$E\left(Y_{1i}|T_{i}=1\right)=S_{1i}\beta_{1}+\sigma_{{\mu \varepsilon }_{1}}\left\{\frac{\theta \left(\hat{Y}\right)}{\varphi \left(\hat{Y}\right)}\right\}\ldots \ldots \ldots \ldots$$

For non-participants with participation:

(13) $$E\left(Y_{2i}|T_{i}=1\right)=S_{1i}\beta_{2}+\sigma_{{\mu \varepsilon }_{2}}\left\{\frac{\theta \left(\hat{Y}\right)}{\varphi \left(\hat{Y}\right)}\right\}\ldots \ldots \ldots \ldots$$

The mean treatment effect on those treated (ATT) is then defined as the difference in expected outcomes between equations (12), (13), which reflect the impact of youth participation in farming (rice cultivation) on farm productivity and welfare participant measures. For counterfactual statements, i.e., if they have chosen to participate in farming (rice cultivation), according to Ref. [50] calculated the expected observed results using Equation (14) and Equation (15), each expressed as:

(14) $$E\left(Y_{2i}|T_{i}=0\right)=S_{2i}\beta_{2}+\sigma_{{\mu \varepsilon }_{2}}\left\{\frac{\theta \left(\hat{Y}\right)}{1-\varphi \left(\hat{Y}\right)}\right\}\ldots \ldots \ldots \ldots$$

(15) $$E\left(Y_{1i}|T_{i}=0\right)=S_{2i}\beta_{1}+\sigma_{{\mu \varepsilon }_{1}}\left\{\frac{\theta \left(\hat{Y}\right)}{1-\varphi \left(\hat{Y}\right)}\right\}\ldots \ldots \ldots \ldots$$

Finally, the difference in the expected results from Equation (14) and Equation (15) is called the Average Treatment Effect on the Untreated (ATU), which is the potential impact of participation on rural youth who did not participate in farming (rice cultivation).

## 5 Results and discussion

### 5.1 Summary of descriptive statistics of variables used in estimations

Table 1 shows the summary of the descriptive statistics of the relevant variables included in the estimate and the mean difference between participants and non-participants in the Youth in farming program (rice cultivation). The data shows that the average age of rural youth is 29.13 years, the majority are male and married (72%), with an average household size of 8. The data in Table 1 shows that most respondents are literate, with an average of 9 school years. This indicates a significant increase in the transition from primary to upper secondary education among rural youth. This may support the FAO's [58] claim that Nigeria's youth literacy rate has increased since 1991 from 66.4% in 2008 to around 80% in 2015. In terms of farm traits, the average farm size cultivated among rural youth is 4.07 ha, suggesting that the majority are small-scale farmers with about 14.04 years of rice-growing experience. Access to institutional services shows that only an average of 31% and 52% respectively received advice from advisors (extension agents) and had access to credit in the past farming season. Similar to our study [59,60], which examines the links between youth in agriculture and access to extension services. The finding of poor access to credit facilities agrees with [61] for youth participation in agriculture in Ghana and [39], for youth engagement in farm business in Kericho County, Kenya. In addition, about 62% and 53% of rural youth, respectively, belong to one or another farmer's association and each had formal agricultural training. The data shows that young rice farmers are on average 2.68 km away from their farms and 14.8 km away from the nearest market where they can sell their farm products. Table 1 also shows the differences between the participants and non-participants in the youth in farm program as they result from the summary statistics of the rural youth surveyed. The results point to a few significant variables, namely that there is a significant difference between participants and non-participants in terms of gender, marital status, household size, farm size, access to extension and credit, membership in associations and formal agricultural training, distance to farm and to the next market. For example, there is a significant mean difference of 2 people between participants and non-participants in the youth in farming (rice cultivation) program in terms of household size. There was also a significant difference of 2.8 ha between participants and non-participants in the area planted for rice cultivation. Likewise, around 59% of the Farm Youth participants have access to extension services, compared to 25% of non-participants.

**Table 1:** Summary descriptive statistics by treatment.

Variable	Full sample (N = 932)		Participants sub-sample (n = 474)		Non-participants sub-sample (n = 458)		Mean difference	t-values	p-value
	1		2		3				
	Mean	SD	Mean	SD	Mean	SD	(2–3)		
Outcome variables									
Yield of rice grain (kg/ha)	5620.24	2514.672	7871.35	1069.339	3290.48	1001.599	4580.87	67.445	0.000***
Per capital food expenditure (₦ ‘000)	26914.04	7460.632	27553.05	8327.886	26252.71	6383.489	1300.335	2.669	0.000***
Per capital total expenditure (₦ ‘000)	39148.83	16522.548	48829.27	15673.618	29130.21	10202.55	19699.055	22.656	0.000***
Other covariates									
Age (years)	29.13	4.101	29.99	3.957	28.24	4.073	1.749	6.655	0.454
Sex (1 = male, 0 = otherwise)	1.13	0.339	1.11	0.318	1.15	0.358	−0.037	−1.657	0.000***
Marital status (1 = married, 0 = otherwise)	0.72	0.449	0.71	0.454	0.73	0.444	−0.020	−0.696	0.104*
Education (Years of schooling)	9.39	3.887	10.58	3.790	8.16	3.596	2.421	9.997	0.692
Household size (number)	8.35	2.383	9.41	2.407	7.25	1.787	2.16	15.515	0.000***
Farm size (hectare)	4.07	2.009	5.44	1.699	2.64	1.120	2.796	29.558	0.000***
Farming experience (years)	14.04	3.557	14.78	3.611	13.28	3.336	1.501	6.586	0.261
Access to extension (1 = yes, 0 = otherwise)	0.31	0.461	0.59	0.493	0.25	0.123	0.571	24.085	0.000***
Access to credit (1 = yes, 0 = otherwise)	0.52	0.499	0.83	0.377	0.20	0.401	0.628	24.653	0.019**
Membership of farmers' union (1 = yes, 0 = otherwise)	0.62	0.486	0.98	0.157	0.25	0.435	0.721	33.863	0.000***
Access to formal agricultural training (1 = yes, 0 = otherwise)	0.53	0.500	0.70	0.459	0.34	0.476	0.346	11.625	0.004***
Distance to farm (km)	2.68	2.230	3.08	2.516	2.26	1.795	0.815	5.590	0.000***
Distance to nearest market (km)	14.80	5.307	15.16	5.525	14.43	5.047	0.733	2.082	0.068*

The *t*-test was carried out to test for difference in outcome and other covariates between of participants and non-participants; *, **, ***represent statistically significant at 10, 5 and 1%, respectively.

Almost 83% of participants in the Youth in Farming program now have access to credit, compared to just over 20% of non-participants. This suggests that the majority of farm youth programs have access to credit facilities compared to non-participants in the program. The membership of participants in the youth in farming (rice cultivation) program in youth organizations is statistically significant and higher than that of non-participants. About 98% and 25% of the participants and non-participants are members of one or the other youth organization. In addition, about 70% of participants in the Youth in Farming program received formal agricultural training in rice agronomy, compared to only 34% of non-participants. These results indicate that participants and non-participants differ systematically. Therefore, rural youth who have received formal agricultural training have proportional advantages over their peers when participating in the Youth in farming program. These results are consistent with [15,62,63], found that improved access to formal education improves participation in the agricultural program.

**Fig. 2:**
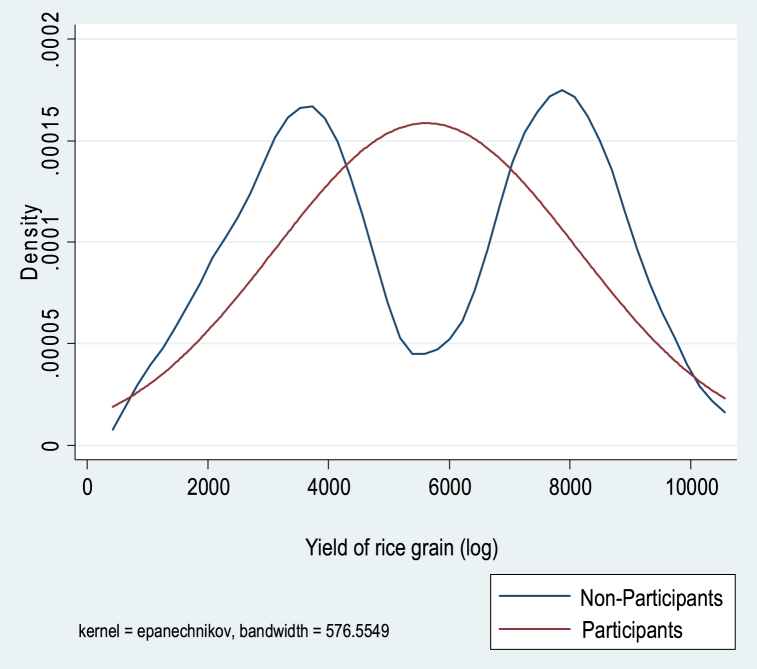
Kernel density estimate of the log of yield of rice grain of participants and non-participants.

**Fig. 3:**
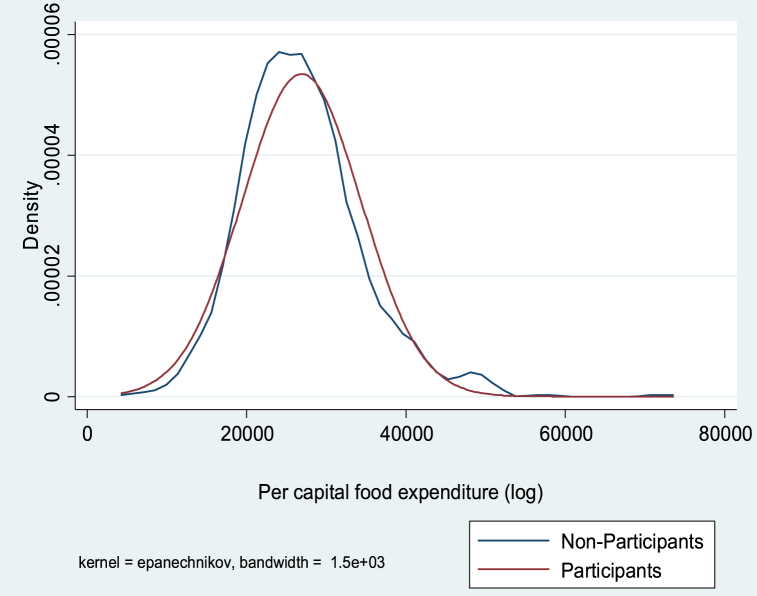
Kernel density estimate of the log of per capital food expenditure for participants and non-participants.

**Fig. 4:**
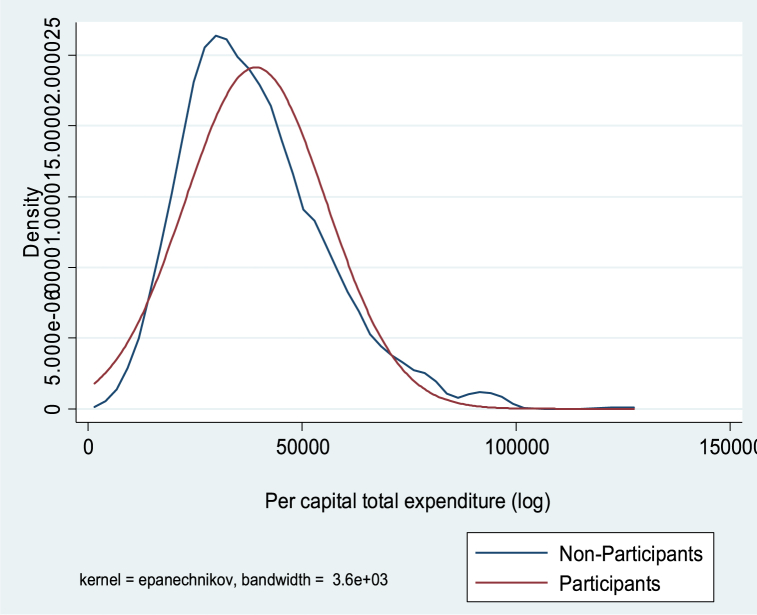
Kernel density estimate of the log of per capital total expenditure (non-food) for participants and non-participants.

Table 1 further shows that the mean difference between participants and non-participants for all outcome variables (rice grain yield, food expenditure per capita and total expenditure per capita) is positive and statistically significant. However, because the mean scores do not account for confounding dynamics, this can skew the impact of participation in farming programs on outcomes of interest. The results in Table 1 cannot be used alone to draw conclusions about the impact of farming program participation on outcome variables. We therefore used the endogenous switching regression (ESR) model as a robust econometric technique to examine the unbiased effects of farming program participation on outcome variables, following the work of [[64], [65], [66]]. Fig. 2, Fig. 3, Fig. 4 shows the kernel distribution showing the variation in skewness between participants and non-participants across outcome variables. Thus, the ESR model proved to be the correct estimate for the study.

### 5.2 Determinants of youth's participation in farming (rice cultivation) program

Table 2 shows the results of the factors influencing rural youth participation in the farming program (rice cultivation) using the Probit model. The results show the probability estimates of the probit model as well as the average marginal effects of youth participation in farming programs. The marginal effect, on the other hand, is more effective than the coefficient to characterize the magnitude of a probabilistic model. This is because the sign and magnitude of the marginal effect determine the direction and magnitude of the potential impact of socioeconomic characteristics on their decision to participate or not [48]. The overall model is statistically significant at p < 0.01 with a chi-square value of 1014.70, implying that youth participation in farming program is strongly associated with socioeconomic, production-related, and institutional variables of rural youth.

**Table 2:** Determinants of youth participation in agriculture program.

Variable	Probit regression		Marginal effects	
	Coefficient	Std. error	dy/dx	Std. error
Age (years)	−0.136***	0.0461	−0.054***	0.018
Sex (1 = male, 0 = otherwise)	−1.134	0.285	−0.381	0.074
Marital status (1 = married, 0 = otherwise)	−0.082	0.273	−0.033	0.108
Education (Years of schooling)	0.103***	0.03	0.041***	0.012
Household size (number)	0.272***	0.052	0.108***	0.02
Farm size (hectare)	0.783***	0.101	0.311***	0.039
Farming experience (years)	−0.049	0.037	−0.019	0.015
Access to extension (1 = yes, 0 = otherwise)	1.834***	0.309	0.593***	0.062
Access to credit (1 = yes, 0 = otherwise)	0.563**	0.215	0.221***	0.083
Membership of farmers' union (1 = yes, 0 = otherwise)	2.223***	0.331	0.725***	0.062
Access to agricultural training (1 = yes, 0 = otherwise)	−0.321	0.24	−0.127	0.094
Distance to farm (km)	0.043	0.044	0.016	0.017
Distance to nearest market (km)	−0.006	0.019	−0.003	0.008
Constant	−2.733	1.009		

***Represent, statistical significance at 0.01 level.

Age has a negative and statistically significant association with youth participation in the farming program at a significance level of 1%, suggesting that the age of rural youth increases the likelihood of participating in the farming program (rice cultivation) by 13.6% reduced. The probability that young people participate in the farming program (rice cultivation) increased significantly with educational level. The partial effect of a one-unit increase in years of education on the conditional probability that youth will participate in farming program (rice cultivation) is 0.103, which means that one additional year of schooling among rural youth results in a 10.3% increase in their participation in rice cultivation. Similarly [35], found that a higher level of education increases the likelihood of young people being engaged in rural agriculture in Imo State, Nigeria. Household size also has a significant effect on youth participation in farming program (rice cultivation), suggesting that farming households with large numbers of family members have an adequate supply of agricultural labour employed to increase rice production be able. According to Ref. [67], large household sizes allow for a sufficient supply of family labour for crop production and the introduction of new agricultural technologies. The likelihood of participating in farming (rice cultivation) increases among rural youth as farm size increases, likely reflecting that a larger farm requires more production resources to handle it. The likelihood of participating in the farming program (rice cultivation) increased by 78.3% for each unit that increased farm size. In addition, access to extension services has a significant impact on youth participation in the farming program (rice cultivation) and this relates to adoption studies that have discovered endogenous expansion of adoption [44]. Non-participants who lack the connection to extension agents to be educated on new agricultural programs could earn less.

Access to credit has a significant positive effect on rural youth participation in the farming program (rice cultivation). This indicates that rural youth who have access to credit are more likely to participate in the agribusiness program. This is because youth with access to credit are better able than their peers to overcome financial constraints and increase their income levels, and access to credit may be a motivating factor for participating in farming programs. The positive association with access to credit is confirmed by Refs. [39,61], for youth participation in agribusiness in Kericho County, Kenya and Ghana, respectively. Among the variables that play an important role in youth's decision to participate in farm business program (rice cultivation) or not is membership in a farmers' association. Membership of a union or association was rated positively and significantly influenced the likelihood of rural youth participating in the farming program. The marginal effect shows that membership in a club increases the likelihood of participating in the farming program by 72.5%. A possible explanation for these results could be linked to the role of social networking and trust between rural youth, which can lead to positive thinking and attitude changes. Our results confirm [34,68], who observed that peers in a social group who are engaged in farming and may be successful may influence the decision to participate in farming.

### 5.3 Impact of youth participation in farming program on farm productivity and welfare outcomes: propensity score matching

**Fig. 5:**
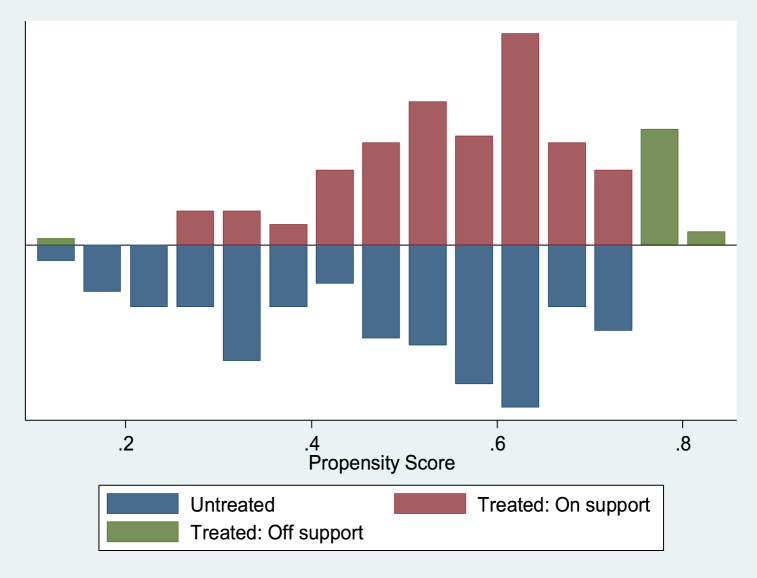
Propensity score distribution displaying common support condition between treated and control cases with outcome variable: yield of rice grain (kg/ha).

**Fig. 6:**
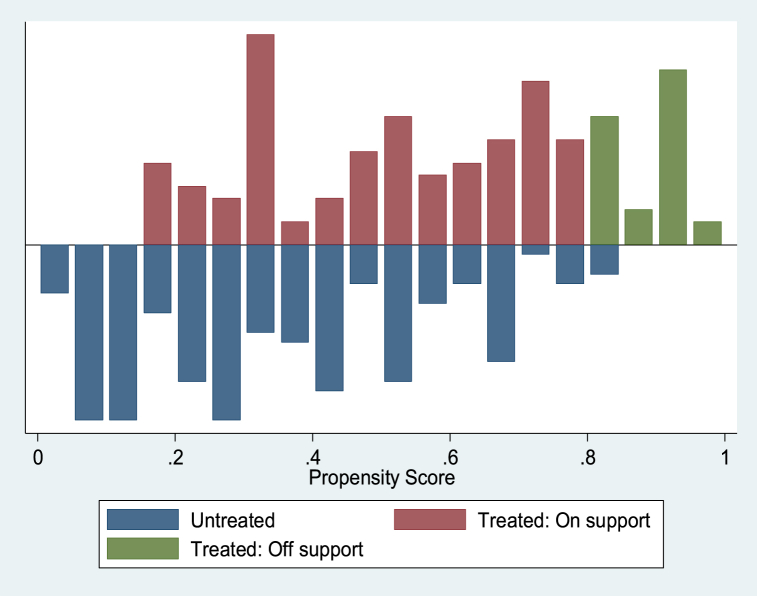
Propensity score distribution displaying common support condition between treated and control cases with outcome variable: per capital food expenditure (₦ ‘000).

**Fig. 7:**
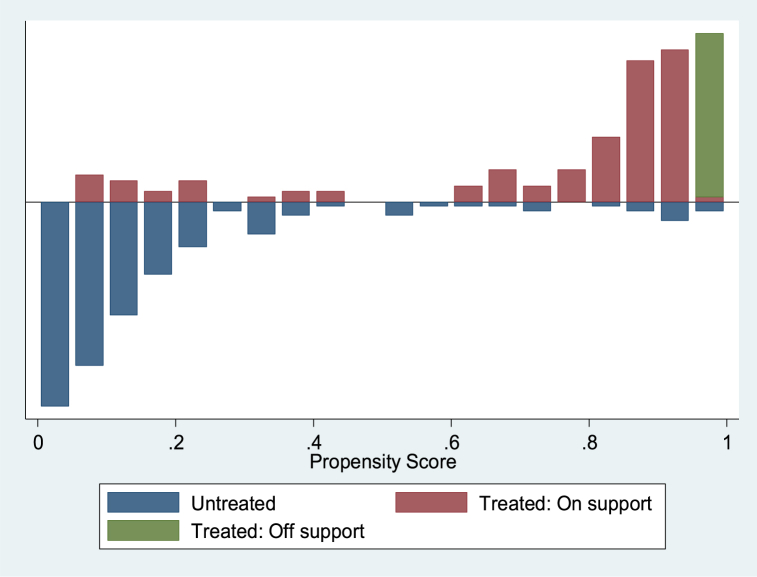
Propensity score distribution displaying common support condition between treated and control cases with outcome variable: per capital total expenditure (₦ ‘000).

Several diagnostic tests were performed to confirm that the matching strategy was consistent and accurate before estimating the impact of participation on the outcome variables using the PSM estimator, which only considers the impact using observables. The distribution of the matching propensity score and the general support condition for the propensity score estimations are shown in Fig. 5, Fig. 6, Fig. 7. The numbers illustrate that the “treated and untreated: support” points to the observations in the participation category that have an appropriate comparison, while the “treated and untreated: support off” indicates the observations in the participation category that lack a relevant comparison. Based on a visual evaluation from the graphic representations of the propensity scores distribution as shown in Fig. 5, Fig. 6, Fig. 7, which reveal a significant overlap in the distribution of participant and non-participant propensity scores, we found the common support or overlap condition checked. This guarantees that there are observations in the non-participants pool that may match the pool of participants.

To further determine the quality of the common support condition, we evaluated the matching strategy for best-fit properties. The test was conducted to ensure that the pre-exposure characteristics of the two groups of youth in farming (rice cultivation) program participants and non-participants were comparable. The results of the balancing property of each covariate used in our analysis are presented in Table 3. Our results show that none of the covariates are significant for the matched samples, indicating that the quality of our match is reasonable for all covariates present in the model. This is consistent with previous research by Ref. [63], who showed that after matching, none of the factors used in the quality test were statistically significant. This implies that the covariate qualities of participants and non-participants were identical. Therefore, the covariate fit tests whether the estimated propensity value adequately fits the observed variables between the participants and the comparison group [69].

**Table 3:** Test of equality of means of variables before and after matching.

Variable	Unmatched Sample				Matched Sample			
	Treated (mean)	Untreated (mean)	% bias	*p* > t	Treated (mean)	Untreated (mean)	% bias	*p* > t
Age	30.02	28.25	440.90	0.000***	29.32	28.57	18.60	0.189
Sex	0.89	0.85	10.90	0.100*	0.85	0.95	−29.60	0.163
Marital status	0.76	0.68	18.20	0.006**	0.70	0.67	7.20	0.441
Education	10.56	8.19	63.70	0.000***	10.01	11.75	−46.70	0.218
Household size	9.42	7.28	100.80	0.000***	8.72	7.10	76.00	0.353
Farm size	5.40	2.63	192.70	0.000***	4.45	4.78	−22.40	0.200
Farming experience	14.74	13.29	41.40	0.000***	14.61	15.04	−12.40	0.191
Access to extension	0.59	0.01	163.00	0.000***	0.40	0.47	−21.50	0.186
Access to credit	0.84	0.26	143.30	0.000***	0.79	0.92	−32.80	0.192
Membership of farmers' union	0.99	0.26	227.90	0.000***	0.98	0.98	−2.50	0.524
Access to agricultural training	0.69	0.33	77.90	0.000***	0.62	0.84	−45.70	0.317
Distance to farm	3.08	2.26	37.30	0.000***	2.84	3.46	−28.40	0.200
Distance to nearest market	15.16	14.43	13.90	0.038**	15.37	19.39	−76.00	0.188

The test of the balance of measured variables between participants and matched non-participants was based on three indicators (pseudo- *R*^2^, p-values of the LR test, and mean standard bias) after conditioning on the propensity scores using two conditioning techniques (NNM and KBM). The results of the overall balance test based on the three indicators are presented in Table 4. After matching with NNM and KBM, the pseudo- *R*^2^ dropped drastically from 80.8% before matching to 24.3% and 40.2%, respectively. This indicates that the systematic differences or biases in the distribution of covariates between participants and those of matched non-participants were significantly reduced as a result of our matching strategy. The LR test for the joint importance of the variables also shows statistically significant differences in measured covariates before matching but not after matching. As a result, we do not reject the hypothesis that the distributions of the covariates are approximately equal after matching between the participants and the non-participants. This indicates that after matching there is no discernible variation in the distribution of the variables between participants and non-participants in the youth in farming program (rice cultivation) after matching. The mean standard deviation has also fallen significantly, ranging from about 71% for the NNM to 86% for the KBM, with the mean bias for the overall covariates being reduced from 87.3 to 32.3 and 28.4, respectively. Given the similar test results between the two matching algorithms, it can be concluded that the match quality is satisfactory, which indirectly satisfies the conditional independence assumption and the need for proper model specification. This assumption states that, after controlling for observable covariates, the assignment of rural youth to farming (rice farming) program participation is “as good as random,” so that the possible results are independent of the participation status.

**Table 4:** Overall quality test of the propensity scores under different matching algorithms.

Status	Matching method	Pseudo R^2^	LR χ^2^	p>(χ^2^)	Mean Standard bias	Total % mean bias reduction
Before matching		0.808	1013.7	0.000***	87.3	
After matching	NNM	0.243	167.88	0.565***	32.3	71
	KBM	0.402	516.35	0.592***	28.4	86

Table 5 shows the estimates of the average participation effects estimated using the PSM based on the nearest neighbor (NN) and kernel (KB) matching algorithms. Using the NN matching algorithm, the causal effect of youth participation in the farming program (rice cultivation) on farm productivity is statistically significant and equal to 4113.51, which is the average difference in farm productivity between participants and non-participants. Our results show that the average treatment effect on treated (ATT) is 4113.51 kg/ha. Assuming that one young people is chosen at random, the average treatment effect (ATE) is 4475.20, which means that farm productivity increases by 4475.20 kg/ha. In terms of welfare outcomes, the causal effect of youth participation in the farming program (rice cultivation) on per capita food expenditure and per capita total expenditure is also significant and equal to 3666.85 and 25696.43, respectively, reflecting the average differences in per capita food expenditures and per capita total expenditures between participants and non-participants. The average treatment effect on treated (ATT) are₦3666.85 and ₦25696.43 for per capita food and per capita total expenditure. In contrast, if a rural youth is randomly selected, the average treatment effect (ATE) would be ₦3666.85 and ₦25696.43 for per capita food and per capita total expenditure, suggesting that he/she per capita food and total expenditure would increase by 3666.85 and 25,696.43 respectively.

**Table 5:** Propensity score algorithms: impact of youth participation on farm productivity and welfare outcomes.

Variables	Parameters	Participants	Non-participants	Difference	S.E.	T-stat
Nearest Neighbor Matching (NNM)						
Yield of rice grain (kg/ha)	Unmatched	7871.69	3295.13	4576.56	69.08	66.25***
	ATT	7875.51	3762.00	4113.51	422.86	9.73***
	ATU	3295.13	4500.17	−1205.04		
	ATE			4475.20		
Per capita food expenditure (₦ ‘000)	Unmatched	27,585.32	26,187.32	1398.01	498.10	2.81***
	ATT	29,730.87	26,064.01	3666.85	2545.18	1.44***
	ATU	26,187.32	18,762.35	7424.97		
	ATE			7753.35		
Per capita total expenditure (₦ ‘000)	Unmatched	48,882.89	29,038.65	19,844.24	888.39	22.34***
	ATT	52,712.11	27,015.68	25,696.43	3790.05	6.78***
	ATU	29,038.65	3478.06	25,560.59		
	ATE			28,811.87		
**Kernel-based Matching (KBM)**						
Yield of rice grain (kg/ha)	Unmatched	7871.69	3295.13	4576.56	69.08	66.25***
	ATT	7871.69	3770.99	4100.70	468.10	8.76***
	ATU	4168.29	4631.04	−462.75		
	ATE			4403.11		
Per capital food expenditure (₦ ‘000)	Unmatched	27,585.32	26,187.32	1398.01	498.10	2.81***
	ATT	27,585.32	25,262.17	2323.16	2972.19	0.78***
	ATU	342.63	5527.48	−5184.85		
	ATE			2973.75		
Per capital total expenditure (₦ ‘000)	Unmatched	48,882.89	29,038.65	19,844.24	888.39	22.34***
	ATT	48,882.89	27,712.05	21,170.84	4772.04	4.44***
	ATU	25,313.16	22,482.07	2831.09		
	ATE			23,876.66		

Based on the results of the KB matching algorithm, our results also show a positive and statistically significant effect on all outcome variables. The results in Table 5 show that the ATT for the participants in the farming program is 4100.70, which means that their participation in the farming program (rice cultivation) contributed significantly to farm productivity with 4100.70 kg/ha. Taking into account the full sample, the ATE is 4475.20 kg/ha. For welfare outcomes, the ATT are ₦2323.16 and ₦21,170.84 for per capita food and total expenditures. In the full sample and considering randomly selecting any rural youth, the ATE are ₦2973.75 and ₦23876.66 for per capita food and per capita total expenditure, respectively.

### 5.4 Estimates of endogenous switching regression (ESR) model.

The Endogenous Switching Regression (ESR) model was used to assess the consistency and validate the results of the PSM. In our estimates, we added two information tools to the selection equation, following [50,70], who used information sources as tools. These are access to agricultural training and access to extension services on youth participation in agricultural programs. Before the implementation of the farming program (rice cultivation), the young people's decision to participate in the program or not can be based on their level of knowledge and knowledge acquired through extension services and training. The relevance criteria lead us to hypothesize that these factors tend to influence rural youth participation in farming programs, but are unlikely to have a significant impact on farm productivity and welfare outcomes (exogeneity criterion). With a simple falsification test according to Refs. [50,70], the validity of these instruments can be assessed. In the selection equation, also referred to as the first stage equation of the ESR model, the instruments are common and statistically significant (*χ*2 = 1.75; *p* < 0.1), (*χ*2 = 10.07; *p* < 0.01), and (*χ*2 = 4.12; *p* < 0.05) for rice grain yield, per capita food expenditure, and per capita total expenditure, respectively.

Our results in Table 6 show that instruments' parameter estimates in the selection equation are both statistically significant, indicating that the instrument relevance assumption is correct. According to the exogeneity hypothesis, the impact of the instruments on the likelihood of youth participating in farming programs (rice cultivation) has an indirect impact on farm productivity and welfare outcomes. Since rural youth's responses to the questions about the instruments were gathered through surveys of the Respondents asking if they had access to extension services and agricultural training in the year before the farm program was introduced, there is less chance of endogenous development, even though this hypothesis cannot be tested generally. According to additional findings of model diagnostics, the connection between the error terms of our outcome equation (farm productivity and welfare) for non-participants and the selection equation is statistically non-zero. This result demonstrates a correlation between the unobservable factors influencing the outcome variables and the factors influencing youth participation in the farming program (rice cultivation). The statistically significant negative correlation coefficient of the error terms of youth participation and rice grain yield and the total expenditure per capita equation for non-participants suggests the practice of self-selection among non-participants. Non-participants likely voted themselves out of participation because they may not have perceived that they would benefit from participation. This result implies that non-participants have lower rice grain yields and lower per capita expenditures than would have been the case for a rural youth population randomly assigned to non-participant status.

**Table 6:** Full information maximum likelihood (FIML) estimates of the endogenous switching regression (ESR) model of Youth Participation in Agriculture (Rice farming), farm productivity and welfare outcomes.

Variable	Selection Equation of Participation	Outcome equation of Yield of rice grain		Selection Equation of Participation	Outcome equation of Per capita food expenditure		Selection Equation of Participation	Outcome equation of Per capita total expenditure	
		Participants	Non-Participants		Participants	Non-Participants		Participants	Non-Participants
Age	−0.139***(0.047)	77.587***(22.975)	−37.109* (22.237)	−0.120** (0.046)	−184.507** (99.978)	50.014 (78.902)	−0.145***(0.046)	−711.094 (2262.91)	−3009.674 (2329.655)
Sex	−1.162*** (0.289)	223.403 (162.836)	70.430 (154.036)	−1.111*** (0.289)	−914.201 (709.796)	−278.878 (550.398)	−1.170*** (0.286)	6722.767 (15966.22)	−22867.46 (16126.05)
Marital status	−0.128 (0.274)	−41.067 (150.641)	147.507 (141.035)	−0.208 (0.277)	−55.727 (657.635)	−192.718 (481.958)	−0.087 (0.269)	−8324.147 (14754.22)	41490.45**(14779.94)
Education	0.107*** (0.030)	3.241 (13.056)	−5.520 (13.782)	0.106*** (0.031)	55.825 (56.796)	−44.482 (48.728)	0.104*** (0.031)	1391.472 (1292.491)	−248.113 (1439.446)
Household size	0.280*** (0.052)	−49.559**(24.263)	−1.427 (29.088)	0.302*** (0.062)	−2876.594***(104.86)	−3010.609***(109.19)	0.273*** (0.051)	−7069.18***(2404.755)	−3388.005 (3045.827)
Farm size	0.767*** (0.100)	−48.269 (38.241)	−7.026 (61.209)	0.735*** (0.097)	270.544** (160.997)	259.584 (253.746)	0.749*** (0.098)	118.874 (3939.038)	−5201.518 (6444.145)
Farming experience	−0.060 (0.037)	−39.50** (20.396)	27.362 (22.471)	−0.058 (0.037)	112.609 (88.997)	81.543** (77.482)	−0.049 (0.037)	4694.327** (1999.17)	2778.288 (2357.267)
Access to credit	0.520** (0.209)	164.475 (142.134)	20.642 (121.286)	0.566** (0.210)	422.130 (612.623)	−239.569 (433.450)	0.533** (0.208)	12414.000 (13963.3)	−3988.43 (12729.54)
Membership of farmers' union	2.286*** (0.332)	−332.943 (466.615)	−229.117* (145.112)	2.195*** (0.321)	3543.508** (1989.849)	−843.570 (675.736)	2.265*** (0.328)	−108635.7** (47415.3))	−13642.25 (15034.38)
Distance to farm	0.032 (0.044)	−14.226 (19.442)	−2.766 (27.669)	0.045 (0.044)	66.832 (85.024)	53.834 (94.536)	0.031 (0.043)	−3611.285** (1905.00)	−3702.088 (2899.291)
Distance to nearest market	−0.003 (0.019)	−15.926* (9.445)	0.464 (10.359)	0.003 (0.020)	−14.151 (41.307)	37.869 (35.619)	−0.0004 (0.019)	−674.628 (924.927)	−237.075 (1085.235)
Access to extension	1.894*** (0.302)			1.987*** (0.309)			1.934*** (0.297)		
Access to agricultural training	−0.292 (0.238)			−0.304 (0.236)			−0.313 (0.241)		
_constant	−2.551** (1.021)	7144.005*** (631.967)	3916.809*** (487.066)	−3.313*** (1.033)	53305.53*** (2692.142)	45267.63*** (1664.07)	−2.461** (1.005)	378795.9*** (64571.87)	333193.3*** (51075.29)
rho1		−0.112 (0.147)			0.354 (0.104)			−0.236 (0.193)	
rho0			−0.207** (0.184)			0.076*** (0.389)			−0.313* (0.235)
LR chi^2^ (2)	1.75			10.07			4.12		
Wald chi^2^ (11)	7.03*			1024.20***			18.78**		
Log likelihood	−7668.6537			−8890.4128			−11845.312		
Number of observations	932	474	458	932	474	458	932	474	458

Robust standard errors in parentheses.

The Wald test of independent equations suggests a joint dependency between the choice and all outcome equations for participants and non-participants, and rejects the null hypothesis of joint independence. We have two different regimes and not just one which matches our specification. This demonstrates the validity of the presumption that the effects of variables are considerably different between the two regimens (with and without participation), supporting the adoption of the ESR model. In all of the ESR model outcome equations provided in Table 6, the difference between youth in farming business participants and non-participants is reflected in the second-stage parameter estimates of the control variables. Parameter estimates of farm productivity and welfare outcome equations between participants and non-participants of the program, indicating the presence of some heterogeneity in the sample. For example, household size, farm size, and farming experience are significantly associated with rice grain yield, per capita food expenditure, and per capita total expenditure, respectively, for participants but not for non-participants. In contrast, association membership, farming experience, and marital status are significantly associated with rice grain yield, per capita food expenditure, and per capita total expenditure, respectively, for non-participants but not for participants. However, variables such as age and household size are significantly related to both participants' and non-participants' results for rice grain yield and per capita food expenditure, respectively. For example, household size has a significantly negative effect on non-participants than on participants when it comes to per capita food expenditure. According to Refs. [50,71], household size significantly lowers consumer spending for both innovators and non-innovators, with the effects on innovators being more pronounced than on non-innovators. The results from Table 6 in the first estimate of the ESR model for all our outcome variables show that young people's participation in farming programs is significantly influenced by age, gender, education level, household size, farm size, access to credit, association membership, and access to extension services. Young people are more likely to participate in farming programs (rice cultivation) if they have access to human, social and financial capital (education, extension services, membership in organizations, access to finance).

Impact of youth participation in the farming program on farm productivity and welfare outcomes: Endogenous Switching Regression (ESR) model.

Results in Table 7 show that the ATE and ATT are nearly the same. The average treatment effect on the treated (ATT) measures the difference between the farm productivity and welfare of the participants and what they would have had they not participated in the farming program (rice cultivation). The average treatment effect on the untreated (ATU), on the other hand, assesses the difference between the outcome variables of non-participants and their counterfactuals. These estimates account for selection bias, in contrast to the mean differences given in Table 1. The ATE shows that yields from youth participation in the farming program increased significantly (p < 0.01) by 4471.53 kg/ha, ₦27131.69 and ₦1862.34, respectively, in terms of farm productivity, per capita food expenditure, and total expenditure.

**Table 7:** Endogenous treatment-effects estimation: ATE and ATT.

Outcome variables	ATE			ATT		
	Coefficient	Standard Error	Estimated t-value	Coefficient	Standard Error	Estimated t-value
Yield of rice grain	4471.53***	208.31	21.47	4133.39***	388.64	10.64
Per capita food expenditure	27131.69***	791.42	34.28	27088.60***	680.31	39.82
Per capita total expenditure	1862.34***	1895.06	9.83	18605.59***	2832.94	6.57

The ATT shows that the impact on farm productivity and welfare of the treated participants has increased significantly by 4133.39 kg/ha, ₦27088.60, and ₦18605.59, respectively, for the results of farm productivity, per capita food expenditure and per total expenditure. Our results confirm recent studies on youth participation in intensive farming; for example [72], shows that youth participation in intensive farming has a positive impact on farm yield and household revenue.

## 6 Conclusion

Using a robust econometric technique in PSM and ESR models, this study examined the impact of youth participation in farming programs (rice cultivation) on farm productivity and welfare. This study finds that age, education, household size, farm size, extension and access to credit, and membership of a social group are determinants of youth participation in farming programs. In terms of impact, the result shows that participants in the Youth in Farming program fare better than non-participants in terms of their farm productivity and welfare status. Similar results from the ESR model's first stage estimation demonstrate that access to finance, association membership, and education are all significantly related to farm productivity and welfare outcomes of participants and non-participants at different levels along the distribution. This indicates the presence of heterogeneous effects dependent on respondents' socioeconomic characteristics, highlighting the need for particular interventions and focusing on particular youth demographics. Several policy implications can be drawn from these results. The government could consider giving young people access to finance, which would encourage their participation in farming programs and agribusiness for the prosperity of the rural economy. In particular, youth-focused loan facilities with low interest rates and flexible payment options can encourage young people to engage in agribusiness activities. Also, the positive and significant effect of membership in social groups requires increased action by appropriate bodies (e.g., agricultural extension agents) who need to increase their efforts to establish and strengthen youth farmer associations in the study area. Increased farm productivity will in turn improve the welfare status of rural youth by increasing per capita food and total expenditures. The results of the study showed a strong correlation between farm productivity, welfare and youth participation in farming programs (through the positive and significant ATT). This therefore underlines the importance of interventions aimed in particular at younger people aged between 15 and 35 who have a great passion for agribusiness and should be encouraged. In terms of limitations, the study was limited to the Northern Nigeria region and specifically to rural young farmers engaged in rice production rather than all farmers. As a result, there is a gap in the literature that can be filled by new studies on the subject.

## Author contributions

Conceptualization, A.K.D., T.A., S.B., S.B.S., and B.A.A; Data curation, A.K.D., T.A., S.B., S.B.S., and B.A.A; Formal analysis, A.K.D., T.A., S.B., S.B.S., and B.A.A; Funding acquisition, A.K.D., T.A., S.B., S.B.S., and B.A.A; Investigation, A.K.D., T.A., S.B., S.B.S., and B.A.A; Methodology, A.K.D., T.A., S.B., S.B.S., and B.A.A; Project administration, A.K.D., T.A., S.B., S.B.S., and B.A.A; Resources, A.K.D., T.A., S.B., S.B.S., and B.A.A; Software, A.K.D., T.A., S.B., S.B.S., and B.A.A; Supervision, A.K.D., T.A., S.B., S.B.S., and B.A.A; Validation, A.K.D., T.A., S.B., S.B.S., and B.A.A; Visualization, A.K.D., T.A., S.B., S.B.S., and B.A.A; Writing - original draft, A.K.D., T.A., S.B., S.B.S., and B.A.A; Writing - review editing, A.K.D., T.A., S.B., S.B.S., and B.A.A.

## Funding

This research was funded by the International Fund for Agricultural Development (IFAD) under the grant 2000001374 “Enhancing Capacity to Apply Research Evidence (CARE) in Policy for Youth Engagement in Agribusiness and Rural Economic Activities in Africa” Project in the International Institute of Tropical Agriculture (IITA).

## Institutional review board statement

The study was authorized by the Institutional Review Board of the International Institute of Tropical Agriculture (IRB/IF-CA/003/2021) for studies involving humans and was carried out in accordance with the Declaration of Helsinki.

## Informed consent statement

Informed consent was obtained from all subjects involved in the study.

## Data availability statement

The data presented in this study are available upon request from the International Institute of Tropical Agriculture (IITA).

## Declaration of competing interest

The authors have declared that no competing interests exist.
